# Prophets With Enchantment: Framing Christian Climate Activism

**DOI:** 10.1111/1468-4446.70061

**Published:** 2025-11-05

**Authors:** Gemma Edwards, Finlay Malcolm

**Affiliations:** ^1^ Department of Sociology University of Manchester Manchester UK; ^2^ Department of Religions and Theology University of Manchester Manchester UK

**Keywords:** christianity, climate change activism, prophetic activism, religious environmentalism, social movement frames

## Abstract

This paper argues for a re‐enchantment of studies of contemporary climate change activism. It focuses upon Christian climate activists in the UK and how they are reinterpreting their theological beliefs in ways that mobilise religious communities. We employ a social movement framing perspective to discover the nature of this ‘interpretative work’ using data from a survey (*n* = 319) and in‐depth interviews (*n* = 62) with Anglicans and Catholics in three church dioceses, a Christian aid agency, and two Christian social movement groups. We found that familiar ‘stewardship’ framings of Christian climate activism dominated in institutional contexts but gave way to ‘prophetic’ framings in Christian social movements. Prophetic framings of climate activism have received very little attention compared with stewardship, but they provide strong theological justification and a distinct emotional inflection to Christian participation in climate protest, and form a bridge to groups like Extinction Rebellion. Prophetic framings were, however, open to prognostic disputes, and remained within an anthropocentric discourse on climate change. With Christians comprising about one third of the world's population, it is of global significance to the environmental movement that in certain enclaves and across denominations, Christian beliefs are being reinterpreted in ways that can lead to their mobilisation not just as ‘climate stewards’ caring for creation, but as ‘climate prophets’ engaged in political protest.

## 1 Introduction

Alberto Melucci described the ‘new social movements’ of post‐industrial society, which included the environmental movement, as ‘prophets *without* enchantment’ (Melucci 1985, 801, our emphasis). The role of the new social movements as carriers of critique and alternative visions fitted well with Melucci's (1985) use of the term ‘prophets’, who in biblical terms act to call out an unjust system and provide more hopeful visions of the future (Brueggemann 2018). While Melucci's work may have revived interest in the role of spirituality in cultural resistance, his prophets were largely without the ‘ritual trappings’ of institutional religious faith (Melucci 1996, 171). This paper argues that we need to turn our attention back to agents of change attached to institutional religious faith, who in the context of the contemporary climate crisis, see themselves as prophets *with* enchantment. In short, we need to ‘re‐enchant’ studies of climate change activism. Here we engage with this task by examining the mobilisation of Christian communities.

Institutional religions like Christianity have an ambivalent relationship to climate change activism (Koehrsen et al. 2022a, 2022b). Judeo‐Christian beliefs, for example, are a key source of the anthropocentrism found in wider western culture, which is often cited as an obstacle to ecological concern (Gentzke and Shipley 2023; Taylor 2010; Hayes and Marangudakis 2003; White 1967). The British Election Study recently indicated that ‘Christians are the least environmentally friendly demographic’, although regular church attendance increased engagement in eco‐friendly practices (Bickley et al. 2024). Treating religion as a survey variable, however, tends to obscure the contexts in which Christians have been engaging with environmental issues since the 1980s. There is a rich history of Christian climate activism in the UK (Nita 2016), and ground has shifted with the upsurge of the global climate movement. Christians were part of the first Extinction Rebellion protests in 2018 (Harmon 2020), and have been a key feature ever since, leading to a resurgence of the ‘political wing’ of Christian climate activism (Nita 2016). In this context, Christians, including members of the clergy, have proven resilient participants in civil disobedience, putting their ‘bodies on the line’ in arrestable actions (Parfitt 2023).

In this paper, we ask how Christians are reinterpreting their religious beliefs in ways that lead to action around climate change. Despite the lacuna that at times has existed between the Sociology of Religion and Social Movement Studies (Peterson 2024; Smith 1996; Hannigan 1991), we suggest that this question can be best examined through the social movement framing perspective. This perspective focuses attention on the meaning making work of activists in the mobilisation process and the resulting ‘collective action frames’ that they construct and communicate (Benford and Snow 2000; Benford 1993; Snow et al. 1986). Whilst it is a mainstay of social movement research, the framing perspective is not without its critics (van Dijk 2023). Here we emphasise framing as an interactional process (Snow and Vliegenthart 2023), embedded within wider ‘discursive fields’ that shape and constrain the meaning making work of activists (Snow 2008; Steinberg 1998).

To examine the interpretative work involved in Christian mobilisation around climate change, we carried out a major study of UK‐based Christian climate activists across two Anglican dioceses, a Catholic diocese, a Catholic aid agency, and two Christian movement groups. Based on survey data (*n* = 319) and in‐depth interviews (*n* = 62) we argue that familiar ‘stewardship’ framings (Nita 2020; Kidwell et al. 2018; Kearns 2011) are open to dispute. ‘Prophetic’ framings (Dear 2022; Cataldo 2022; Brueggemann 2018; Bock 2016; Slessarev‐Jamir 2011; Gutterman 2006) instead dominated in the political wing of Christian climate activism, providing theological justification, moral impetus, and a distinct emotional inflection to Christian participation in political protest. These findings challenge the leading view in the current literature (Bomberg and Hague 2018; Kidwell et al. 2018; Nita 2016), carried out in the years prior to the establishment of Extinction Rebellion, which prioritises stewardship framings over the fast‐growing prophetic frame, which we found to be galvanising political forms of Christian climate activism.

We first provide the context of ‘Green Christianity’, before outlining the framing perspective and methodology in more detail. We then turn to an analysis of the data, where we discuss the stewardship framing and diagnostic and prognostic disputes amongst our sample. We argue that Christian social movement groups forged an alternative ‘prophetic framing’ where they effectively harnessed the Christian prophetic tradition to mobilise and bridge to groups like Extinction Rebellion. We then consider how these framings related to actions and emotions. We show that prophetic framings were also open to prognostic disputes, and conclude that, like stewardship, they were embedded within a broader anthropocentric discourse on climate change.

## 2 Green Christianity

Religion entered the debate over the environment in the 1960s with the claim that it was partly to blame for the ecological crisis (Kearns 1996, 55). White (1967) argued that Judeo‐Christian beliefs about humans as ascendant over, and separate from nature, were an established part of western cultural understandings about human and non‐human relations. These beliefs were rooted in the biblical story of Creation, where in Genesis 1: 26–28, humanity is given ‘dominion’ over nature to use it for its own purposes. Such beliefs are anthropocentric (human‐centred) and were therefore seen as an obstacle to the development of ecological concern (White 1967). Taylor (2010), for example, argues that anthropocentric beliefs are the reason why Christianity has struggled to develop ‘dark green’ religion. Other aspects of Christian belief have also worked against ecological concern, such as the apocalyptic belief that God will destroy the current world and create a new one (Veldman 2019). Such thinking prioritises ‘saving souls’ over ‘saving creation’ and has been found to be prevalent amongst Evangelical Christians (Kearns 2011, 420). Existing research suggests that these anthropocentric and end‐times orientated world views demotivate climate change concern (Michaels et al. 2020; Hope and Jones 2014).

If religion was part of the cause of the ecological crisis, then it could also be an important part of the solution (Gottlieb 2006; White 1967). Eco‐theology sought to make it such by critically displacing the belief that humans have a God‐given dominion over nature (Deane‐Drummond 2008). Instead, as Nita (2020, 157) argues, eco‐theologians reinterpreted the biblical stories of Genesis to produce an alternative ‘stewardship model for environmentalism’, which emphasises human responsibility to care for nature as part of God's creation (Peterson 2024, 126). ‘Green’ Christianity has to some extent therefore revised the traditional story to motivate climate concern (Nita 2020, 166). This has been reflected in Church responses to environmental destruction and climate change. In 1990, the Anglican Church added a fifth mark of mission to ‘strive to safeguard the integrity of creation’. In 2006, Christian conservationists A Rocha launched the ‘Climate Stewards’ programme to encourage Christians to reduce their carbon footprint, followed by ‘Eco Church’ in 2015 to support Churches to reduce their carbon footprint and make lifestyle changes as part of caring for creation. Christian efforts to respond to climate change also received a significant boost with Pope Francis' publication of the encyclical *Laudato Si’* on 24^th^ May 2015. This public letter called upon Christians to respond to climate change and the ‘Cry of the Poor and the Cry of the Earth’ by engaging in ‘care for our common home’ (Francis 2015). In 2019, the Church of England set a 2030 target for carbon net‐zero, the earliest date set by an institution in the UK.

These changes in Church institutions have been forced through by the campaigning pressures of Christian activist groups. Nita's ethnographic study of Christians in the UK climate movement (2005–15) argues that Christians have played an important role since the 1980s (Nita 2016, 28), with groups like Christian Ecology Link (1982, later renamed Green Christian) (Nita 2016, 32), GreenSpirit, and the anarchist group Isaiah 58. In 2004, Green Christian launched ‘sister organisation’ Operation Noah as its campaigning wing with a focus upon Church land use and divestment from fossil fuels. Christian Climate Action, a social movement employing nonviolent direct action, emerged in 2012 (Williams 2020). Members were actively involved in the wider climate movement, including anti‐fracking protests which saw some members arrested (Harmon 2020). Christian Climate Action has grown since 2018 through working with Extinction Rebellion, positioning themselves as ‘the Christians in Extinction Rebellion’ in the UK and internationally (Harmon 2020). Some members are also variously involved in Insulate Britain and Just Stop Oil. Alongside these activist groups, Christian aid organisations (e.g., Christian Aid, The Catholic Agency for Overseas Development [CAFOD], The Salvation Army, and Tearfund), have turned their anti‐poverty social justice work to campaigning around environmental issues and the impacts of climate change.

## 3 Framing and Mobilisation

The framing perspective is a useful one to employ in an examination of Christian climate activism, where activists are grappling with how to interpret situations of climate change in ways that can mobilise Christian communities. The concept of framing captures the interpretative and meaning making work that activists do to construct and communicate meanings of situations in ways that lead to mobilisation (Benford and Snow 2000). The resulting ‘collective action frames’ produced through these interactive processes can be thought of in Goffman's (1974) terms as ‘schemata of interpretation’, or a pattern of interpretation that is employed to render reality meaningful and supply an answer to the question ‘what is going on here?’ (Benford 1993, 678). Collective action frames, as opposed to the interpretative frames usually employed to understand situations (such as media frames), have three main tasks: to provide a ‘diagnostic’ framing of the situation, which produces a shared interpretation of the problem and makes attributions as to who is to blame (Benford and Snow 2000, 616); to provide a ‘prognostic’ framing of the situation which produces a shared interpretation of the solutions to the problem and what should be done to achieve them, including strategies and tactics (Benford 1993, 699); and to provide motivation for action, a shared interpretation of why people should act now and why collective action would be efficacious (Snow et al. 1986).

Through the various framing tasks, the purpose is for activists to interpret reality in ways that are alternative to, and challenge, extant meanings (Benford and Snow 2000, 614; Gamson 1992). Activists must also align their collective action frames (their alternative way of interpreting the situation) with the existing meanings, ideas, beliefs and values of potential supporters and participants. They do this through a range of ‘frame alignment processes’ including transformation (changing) frames, bridging between distinct frames, extending existing frames to include new issues, and amplifying or clarifying the values or beliefs of existing communities (Snow et al. 1986). Faced with the problematic situation of climate change, Christians have been negotiating shared interpretations of the problem, attributing blame, articulating alternatives, and urging others to act (Benford and Snow 2000, 615). They have done so by amplifying, extending and transforming theological beliefs in efforts to align climate change concern with extant Christian beliefs. Christian framings of the problem, and the remedies for it are however not unified, as found in studies that emphasise religious environmentalism as an ‘embattled terrain’ (Koehrsen et al. 2022a, 2022b). This is also the case in the global climate movement (Svensson and Wahlström 2023; Della Porta and Parks 2014; Wahlström et al. 2013). In these situations, interactive processes to frame problems, make attributions of blame, propose solutions and strategies to achieve them, are contentious and disputed. Following Benford's (1993) identification of diagnostic and prognostic frame disputes, we focus here on interpretative framing processes, the ways in which they are contested and differ across Christian groups, and the consequences for Christian climate action.

## 4 Methods

The dataset comprises 319 survey responses, and 62 semi‐structured interviews with UK‐based Christian climate activists, collected in 2023‐4 as part of the ‘Religion, Theology and Climate Change’ Project (AHRC). To capture the variety of Christian climate activism, we sampled Christians working on climate issues across denominations and in three main contexts: church dioceses, Christian aid agencies, and Christian social movements. We included two church dioceses in the north of England (Manchester Church of England Diocese and Salford Catholic Diocese), and one in the south (Oxford Church of England Diocese), where there was significant activity around creation care, such as carbon net‐zero projects, Eco‐Church, and The Guardians of Creation project. We included one Christian aid agency, the Catholic Agency for Overseas Development (CAFOD). CAFOD work with developing countries delivering aid, and therefore deal first hand with the negative consequences of climate change. In terms of Christian movements, we included the social movement Christian Climate Action (CCA), which operates within Extinction Rebellion. We also included the Christian climate campaigning organisation ‘Operation Noah’, who run the ‘Bright Now’ campaign for Church of England divestment from fossil fuels, and petitions the Church of England over its land use. Participants drawn from each of these groups were Christians who were in some way active in tackling climate change and covered a broad spectrum from those operating within their local church and diocese, to those employed in climate‐related roles within CAFOD or campaign organisations, to grassroots movement activists. Participants in the study sometimes cut across these different groups; for example, participants from Church dioceses were sometimes also members of CCA, and participants of CCA were sometimes also members of Operation Noah.

Surveys were distributed to the six groups as a purposeful sample (Palinkas et al. 2015) and administered through Qualtrics. Survey data were analysed using SPSS v29. The survey asked about climate‐related theological beliefs through a series of statements to which respondents indicated their agreement (on a Likert scale, 0–10), and about feelings around climate change and justified actions to combat climate change, as well as religious and secular influences on ecological views. 319 responses were gathered between May and December 2023. There was an uneven spread of respondents from across the organisations (CAFOD, 94; Oxford Diocese, 89; CCA, 93; Salford Diocese, 46; Operation Noah, 39; and Manchester Diocese, 30). However, since respondents could select multiple options for their affiliation, an aggregate of these numbers is higher than the total responses (319).

The survey response rates capture a high proportion of the overall population. It is estimated that Christian Climate Action have around 300 volunteers, giving us nearly one third of the population. Operation Noah is a small organisation, and we have captured responses from most of their staff or volunteers. Those working with environmental issues in the three church dioceses are also relatively small, encompassing a restricted group of environmental officers, ministers and volunteers. We expect to have gathered responses from most of the population in those three organisations. CAFOD is a mid‐sized charity and so 94 responses across volunteers and salaried staff suggests a high response rate.

Given that we were working mainly with Catholic and Anglican groups, these religious affiliations were the most predominant: 167 said they were Anglican/CofE, 128 Roman Catholic, 34 Other Christian, and 17 Other Religion. There is less age and ethnic diversity^1^ in the sample. Most of the respondents fell into the older age brackets: 68% above 55‐years old, and 5% below 34‐years old. 55 per cent were female and 42% male. The vast majority were ethnically white (84%), whilst 6% were non‐white, and 10% left their ethnicity unspecified. Most were educated to tertiary level (87%), with 40% having a university Bachelor's degree, and a further 47% having undertaken further study at Master's or PhD level. Most of the respondents identified as heterosexual (88%) and had children (69%). Respondents were also asked, on a scale of 0–10, where they would place their political views, with “0” indicating “left” and “10” indicating “right”. These results were grouped into “Left” (0–3), “Centre” (4–6), and “Right” (7–10). Half were Left, a quarter were Centre, and 6% Right, whilst the remainder preferred not to say.

Interviewees were recruited through a combination of the survey and gatekeepers. Interviews discussed a person's background in Christianity and environmentalism, how they got into climate activism, what they do and why. It also discussed their climate‐related theological beliefs (using the survey results on agreement and disagreement with various climate‐related theological beliefs as a prompt), the influences on their theological views and climate change, and perceptions on the role of Christian institutions. Interviews were spread evenly across the groups (CAFOD, 10; Oxford Diocese, 11; CCA, 11; Salford Diocese, 10; Operation Noah, 10; Manchester Diocese, 10). 36 interviewees were Anglican/CofE, 25 Roman Catholic and 7 were other Christian or other religious. 38 were female, 23 male, and 1 non‐binary. 34 were above 55‐years old (54%), and 9 below 34‐years old (14%). 51 were ethnically white, 8 belonged to another ethnic group, and 3 preferred not to say. 38 per cent had at least a university Bachelor's degree, and a further 60% had also undertaken postgraduate study at Masters or PhD level. 60% identified as “Left”, 16% were “Centre”, and 3% were “Right”, while 21% preferred not to say. Interviewees are referred to here using their interview number to retain anonymity.

All 62 interviews were transcribed and analysed using NVivo 12 by two members of the project team, from January to April 2024, following a process of thematic analysis (Braun and Clarke 2006). Initial coding used a deductive framework based on the survey questions with 7 top‐level codes and 93 sub‐codes, with a further 17 codes added inductively. Using the survey and interview data in combination we were able to analyse the nature of participants' theological beliefs and emotions regarding climate change, and how their Christian beliefs figured in their accounts of participation in various forms of climate activism.

## 5 Findings

### 5.1 Stewardship Framing

Stewardship was a dominant way of framing Christian climate activism among our participants, although as we will argue, it was not without contest and in Christian social movement groups it was giving way to an alternative prophetic frame. The survey data showed widespread agreement with the statement ‘humans are given responsibility to steward God's creation’. All groups scored a mean average of 9.3 or above out of 10 (where 10 denotes complete agreement). Interpreting Christians as ‘climate stewards’ with a responsibility to care for God's creation was therefore widely accepted. Stewardship was referred to as a kind of ‘default language’ (3001, CCA), because ‘apart from anything else, it's useful’ (6002, Oxford Diocese). Stewardship has been useful for example in the task of reframing traditional Christian beliefs about the relationship between God, humans, and nature by rejecting the idea that humans had ‘dominion’ over nature, or as one interviewee put it, ‘you know, rule and conquer and subdue, tame the world, it's all God‐given, let's take whatever we can from it’ (6014, Oxford Diocese).

Whilst stewardship ideas existed across the sample, understanding Christian climate activism in terms of stewardship was the primary frame of interpretation we found operating in church dioceses and CAFOD. 94 entries were categorised under the ‘stewardship’ code, with 70 of these within the church dioceses and CAFOD and 24 within the social movements groups, Operation Noah and CCA. However, much of the discussion of stewardship coded within interviews with CCA and Operation Noah members was concerned with criticising the diagnostic and prognostic aspects of the frame, which we explain below. Amongst the other groups, stewardship and related ideas of ‘creation care’ were seen as a ‘very effective way’ to talk to people about why climate issues should be relevant to their faith, especially young people (6008, Oxford Diocese), and one that was believed to have strong theological support: ‘I do believe the book of Genesis makes it clear that our role is to be stewards of God's creation’ (1003, Manchester Diocese). As such, participants argued that:

I want to use the language of stewardship. It just does seem to be the case that God has made the world in such a way that human beings have got an awful lot of power that other creatures don't have, and I think they have a responsibility to use it (6017, Oxford Diocese).

This is not to say that stewardship was always employed uncritically within church dioceses and CAFOD. Participants at times picked up on the debate over stewardship in ecotheology and Christian activist circles (Valerio 2021; Kearns 2011), disputing the ‘slightly feudal power dynamics’ of the word (6015, Oxford Diocese), and how the notion of humans stewarding nature implies that ‘we're over and above and better than and separate to creation’ (4018, CAFOD), which was described as ‘a kind of superhero Marvel‐esque idea’ of the role of humans in relation to nature (4017, CAFOD).

In the social movement groups of CCA and Operation Noah however, major disputes arose over elements of the stewardship frame. Firstly, there were disputes over diagnostic aspects, primarily the ‘attributional orientation’ of the frame (Snow et al. 1986, 474), in terms of *who is to blame* for climate change. As Snow and colleagues argue (1986, 474), the identification of an injustice is not enough on its own to mobilise protest ‐ it must also become attributed to ‘system failings’ rather than individual failings. Christians in the social movement contexts levelled blame not at individuals, but at governments and corporations who comprised a ‘global economic system that is really unjust and just uses people and the earth for capitalist means’ (3003, CCA). In CCA, this shift in blame attribution was often expressed through citing the biblical example of Jesus turning over the tables of the money lenders:

If Jesus goes into the temple and turns over the tables of the money changers, is he acting directly against that particular money changer, or is he acting against a system of domination, of extractivism against the people, the poor people of the system? No, it's against the system (1002, CCA).

Disputes also followed regarding the prognostic aspects of the stewardship frame, in terms of the question ‘what is to be done’ (Benford and Snow 2000). Stewardship framings provided an understanding of Christian climate activists as ‘climate stewards’ with a personal responsibility to nurture God's creation. This produced a focus upon sustainable lifestyle and personal transformation strategies (Kidwell et al. 2018; Nita 2016). In church dioceses, many participants were involved in Eco Church to reduce their carbon footprint and improve the sustainability aspects of Church life, and in initiatives focussed on personal and lifestyle change, like recycling, rewilding, community gardening, solar energy, and zero carbon. Participants in the social movement groups questioned the utility of strategies employed to ‘steward’ God's creation and what Kearns (2011, 426) has referred to as their ‘managerial approach’ in the sense that ‘it's one of our programme priorities that we've got our eco church, you just need to sign up for this, get a badge. Well that's not good enough’ (3001, CCA). Such lifestyle forms of climate activism were critiqued for being ineffective:

We are not going to recycle our way out of the climate crisis, we really aren't […] you need to have someone who is going to keep setting it in the bigger context so that people don't just think, if I get Eco Church gold [award] then I've sorted the problem out (3004, CCA).

The prognosis offered by stewardship in response to climate change—lifestyle activism and sustainability—was therefore disputed. Participants in CCA and Operation Noah criticised these strategies for failing to see the necessity of changing a wider system. Participants argued that ‘it would be like saying ‘“do you know you can have a wild garden?” it's better than nothing […] but the system's still shit’ (3003, CCA), or ‘we're fiddling around with bug hotels and we're not tackling the systems, the [in]justice’ (3011, CCA). Instead, ‘you realise that it is not good enough just to look at your own footprint and you need to campaign for transformational change’ (2016, Operation Noah):

We have moved from the idea that all this requires is personal action into a clear acknowledgement that actually there are social forces, political forces and very powerful vested interests (3002, CCA).

In shifting the focus to system‐level change, Christian social movement groups engaged in frame transformation (Snow et al. 1986, 473): ‘good stewards’, it was argued, must look to the root cause of problems and target them with strategies that address the systems that are really to blame. A member of CCA articulated this transformation of the stewardship frame when they stated that, ‘I think stewardship for me, it includes *challenging the systems that are wrong*’ (3003). Subsequently, in the social movement groups we found a different framing of Christian climate activism growing in prominence: a ‘prophetic frame’, which centred on the Christian responsibility to take up the fight against systemic injustice.

### 5.2 Prophetic Framing

Like stewardship, notions of social justice in relation to climate change also received widespread support. Survey data showed strong agreement with the statement that ‘those who are least culpable for the climate emergency—the global poor and future generations—will be most impacted by it’ (mean average of 9.5 out of 10 in every group). Whilst a commitment to justice in the context of climate change co‐existed with stewardship across the sample (see also, Kidwell et al. 2018), it became the dominant way in which Christian movement groups framed the issue of climate change and its relevance for Christian activism. The links between justice and Christian responsibility to care for creation were regularly made by our participants: ‘justice and peace, and integrity of creation, are not two separate things’ (2011, Operation Noah). Using justice as their starting point, they interpreted the Christian responsibility to act on climate change not primarily through the frame of stewardship, but through the frame of the Christian prophetic tradition.

The Christian prophetic tradition relates to Biblical scriptures in which prophets deliver God's message of truth and justice to oppressive rulers through means of nonviolent resistance. By invoking the prophetic tradition, Chrsitian activists highlighted the influence of prophetic exemplars from the Bible such as Jeremiah, Ezekiel, Isaiah and Jesus, to critique unjust power structures and present a compelling alternative vision. Brueggemann (2018) writes that the biblical ‘prophetic imagination’ involves, firstly, critiquing the dominant order of an unjust society by dismantling the dominant consciousness and delegitimising the present order; and secondly, ‘futuring’, not so much telling the future, but creating alternative visions of the future, an alternative consciousness that ‘serves to *energize* persons and communities by its promise of another time and situation towards which the community of faith may move’ (Brueggemann 2018, 3, original emphasis).

This prophetic imagination is brought to bear on the climate crisis in John Dear's updated (2022) version of *The Sacrament of Civil Disobedience,* originally written for the Plowshares Movement (Anne and Montgomery 1987). In it, Dear and his collaborators announce a revival of the prophetic tradition in the context of climate change, with the editors arguing that ‘Christians are hearing the call anew to step up to the plate and confront governments with the radical action that is required, if we are not to condemn practically all life on earth to extinction’ (Donald and Parfitt 2022, *p*. x‐xi). This radical action, according to Dear, involves forms of nonviolent direct action characteristic of biblical prophets, who often suffered significant personal costs for delivering their messages of truth and justice. Many members of CCA had participated in a book club devoted to the discussion of Dear's book.^2^

Whilst prophetic framings of Christian social justice activism are far from historically *new* (Cataldo 2022; Bock 2016; Slessarev‐Jamir 2011; Gutterman 2006), we found a *renewed* attempt to frame Christian climate activism in prophetic terms. During interview analysis, we attributed 72 items to a distinct ‘prophecy’ code. Presenting Christian climate activism in ‘prophetic’ terms was evident in interviews with 80% of participants in Operation Noah across 20 code items, and 100% of participants in CCA across 38 items. This compared to 20% of participants in the case of Salford (*n* = 2 codes) and CAFOD (*n* = 4 codes), and 30% of Oxford (*n* = 3 codes) and Manchester (*n* = 5 codes). A total of 58 of the 72 codes appeared across just CAA and Operation Noah interviews, and where it appeared within the Diocese of Manchester, those participants also identified as CCA members. Moreover, within these interviews, the prophetic frame was viewed almost entirely positively. For the social movement activists, it was clear that ‘Christians have got this prophetic voice, this accountability voice and it's really quite obvious I think’ (2008, Operation Noah).

The prophetic voice, like the biblical prophets, was a voice ‘calling out from the margins’ (2005 and 2008, Operation Noah) about an unjust system which was moving us towards climate catastrophe. This framing also drew mobilising power from amplifying the existing Christian beliefs in social justice (Kearns 1996, 57). Particularly in Catholicism, Christians have a clear tradition in linking social justice for the poor with biblical prophecy and political activism aimed at system critique, as seen in Latin American Liberation Theology (McGovern 1989, 69–70). Indeed, the phrase ‘cry of the earth, cry of the poor’ was proposed by a Brazilian liberation theologian (Boff 1997), and was taken up by Pope Francis in his encyclical, *Laudato Si’* (Francis 2015, #49). Through the prophetic framing, participants in social movements saw their role as ‘climate prophets’, who were ‘the grit in the oyster’ (2002, Operation Noah), and whose task it was ‘to be disruptive to get people's attention’ (3010, CCA). As a participant from CAFOD put it, there are those who are called to be the ‘rufflers’:

In the Old Testament, prophets really ruffled feathers so perhaps we should be doing a bit more ruffling, but I don't think I'm called […] to be a ruffler. I don't think I am at the moment, but things could change (4009, CAFOD).

### 5.3 Frames, Actions and Emotions

To call the unjust system to account, climate prophets were compelled to engage in different kinds of actions compared to climate stewards. These actions involved ‘speaking truth to power’, a key characteristic of the prophetic activist in other social justice movements (Slessarev‐Jamir 2011). Truth telling actions were employed to puncture and delegitimise the dominant order and open space for an alternative consciousness (Brueggemann 2018). Speaking truth to power was therefore a form of prophetic Christian ‘witness’:

What is the point of the church at this time […] if we can't be prophetic now? The world is crying out for people to tell the truth […] if you just look at the Old Testament prophets, half of the Bible is these prophets speaking truths of their generation (2016, Operation Noah).

Speaking truth to power took the form of various kinds of climate action, including political campaigning, demonstrations, protests, public prayer and vigils, and nonviolent civil disobedience. Tactics of direct action and civil disobedience gained strong theological justification, especially in CCA under the influence of Dear (2022). Jesus was described as ‘basically quite a nonviolent direct action demonstrator’ (3003, CCA) and ‘a radical who spoke truth to power and was executed because of it’ (3004, CCA). Jesus provided the model for strategies of nonviolent civil disobedience. In one sense, answering the question ‘what is to be done?’ as a climate prophet was simple: ‘what “would Jesus do?” I'm pretty sure he'd be on the streets' (3002, CCA). Prophetic Christians in other historical social movements, from American civil rights to the peace movement, also provided models of action. The climate prophet framing in this respect achieved what Benford (1993, 693) refers to as ‘narrative fidelity’: it was able to resonate with a prominent version of the culture and story of Christianity when it came to participation in social justice activism.

The prophetic framing thus amplified beliefs about the necessity of Christians to undertake nonviolent direct action in the face of systemic injustice, and supported participation in civil disobedience, with seven of our interviewees arrested for actions like road blocking, glueing on, slow marching, property damage, and disrupting transport systems. The argument in CCA was not only that these kinds of tactics could be theologically justified, but that civil disobedience could be an act of embodied truth telling—a moral act through which one performed God's justice and showed faithfulness to God, even if it came at personal cost. As one member of CCA put it:

Sacrifice is at the heart of our faith. Jesus on the cross is our central image, so helping people to see that sacrifice is something that we're really called upon in this extreme situation (3005, CCA).

**FIGURE 1:**
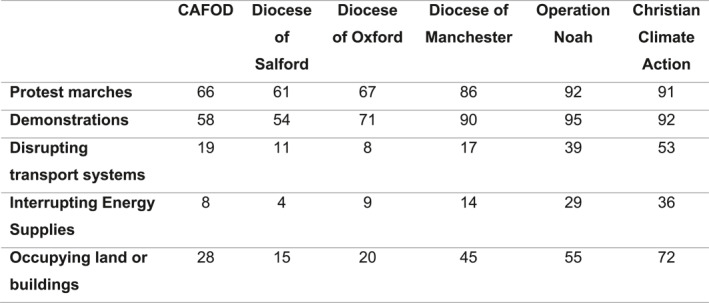
Which actions do you think are justified in the effort to reduce the effects of climate change? All protest‐related answers (%). *Source:* ‘Religion, Theology and Climate Change’ Survey, 2023.

There was evidence from the survey to suggest that in the groups where prophetic framings dominated (CCA and Operation Noah), higher levels of justification were therefore found for political protest actions like marches and demonstrations, and for stronger forms of direct action (see Figure 1).

**FIGURE 2:**
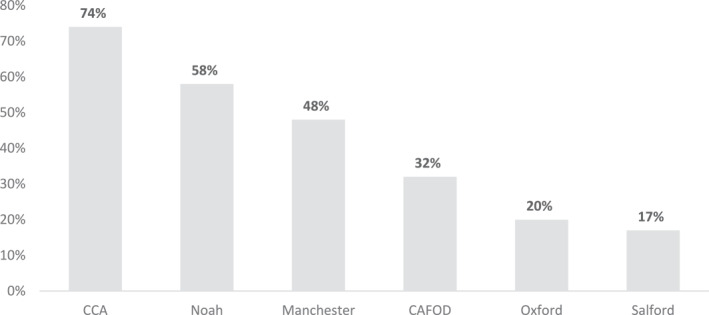
Stronger actions by group. *Source:* ‘Religion, Theology and Climate Change’ survey, 2023.

We further investigated which groups were more likely to see the stronger actions (disrupting transport systems, interrupting energy supplies, and occupying land and buildings) as justified. In total, 41% of respondents selected *at least one* of these stronger actions. Figure 2 shows how that breaks down by group. It shows that in groups dominated by the prophetic frame (CCA and Operation Noah), respondents were significantly more likely to say that at least one of the stronger actions was justified.

It is clear to see therefore how the prophetic framing provided something of a natural ‘frame bridging’ (Snow et al. 1986, 467) to groups like Extinction Rebellion. Extinction Rebellion also compels participants to ‘tell the truth’ through nonviolent civil disobedience and has been described as a secular form of Brueggemann's prophetic imagination (Williams 2021). It is important to note however that frame bridging to groups like Extinction Rebellion was not one‐way. Prophetic framings may have aligned with Extinction Rebellion to mobilise Christians in some cases, but others found a *bridge back* to Christianity through participation in the secular movement. Principles of love, nonviolence, and the very language of Extinction Rebellion were said to ‘resonate so strongly’ (3002, CCA) with Christian beliefs and values. In the context of the wider climate movement:

There's such a wonderful diversity of people who are united in caring for the Earth, in love and justice and truth, and all those things which are qualities of the kingdom of God. And it's much more real and tangible in that setting than it ever has been in church, which is shocking, and weird, and beautiful (3011, CCA).

Like in the global climate movement, however, prognostic framing—the understanding of ‘what should be done’—was rarely without dispute (Svensson and Wahlström 2023; Wahlström et al. 2013). Tactics of civil disobedience were disputed by some members of CCA and Operation Noah on account of effectiveness. One participant (2007, Operation Noah) questioned whether stronger actions were ‘bringing the public alongside’, and if not, are they ‘causing people to turn away from caring or doing stuff’. The tactics were also disputed on grounds of whether self‐sacrifice was a moral obligation for Christians, arguing that it is only one of the Christian values that could be amplified: ‘there are other Christian callings, like we're called to community and family and that kind of thing’ (3002, CCA). Another participant from CCA expressed guilt at the impact of their actions and felt ‘really uncomfortable with the blocking of the roads. I remember cycling home after that protest and seeing hundreds and hundreds of people trapped in traffic’ (3008, CCA). As an alternative, and in line with their faith, they had been increasingly engaged in silent vigils and public prayer, saying that ‘it's not a normal, noisy protest, but for me that's where I've located my activism’ (3008, CCA).

Differences in prognostic framing were also evident between CCA and Operation Noah. In Operation Noah, much of the prophetic ‘speaking truth to power’ work involved campaigning in partnership with the organisations they were seeking to reform. Operation Noah campaigned for over a decade partly from within the Church of England to influence its eventual fossil fuel divestment decision in June 2023.^3^ So, in addition to truth telling acts of civil disobedience found in CCA, groups adopting a prophetic framing also rooted solutions in political campaigning. Operation Noah's funding structure might partly explain the different emphasis. As an organisation with charitable status they are more restricted in the political action they can take (see Nita 2016, 214),^4^ compared with CCA who are funded through individual donations. Funding structures may also explain why CAFOD affirmed the prophetic frame far less strongly than their investment in campaigning and liberation theology might otherwise suggest.^5^ As the official development agency of the Bishops Conference of England and Wales, CAFOD also have charitable status and their work is funded through donations from parish churches and the UK Government. The degree of independence from institutional financial support and charitable status appear to be important factors shaping the adoption of prophetic framings (those most critical of systems and institutions), and the extent to which more disruptive truth telling tactics (such as civil disobedience) were proposed.

**FIGURE 3:**
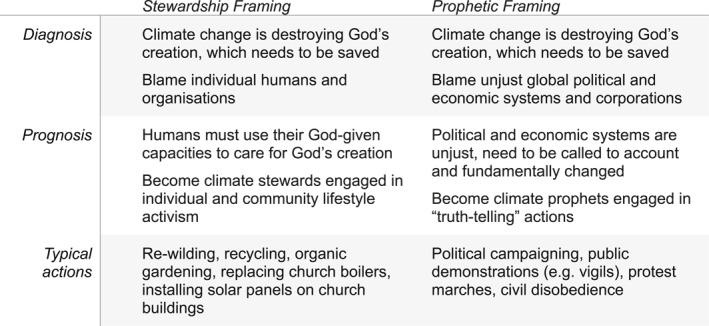
Diagnosis, prognosis and action.

We summarise the prophetic frame and the stewardship frame, and the range of strategies and actions they encompass, in Figure 3 below.

We also found differences in emotions between groups attached to prophetic framings compared with stewardship framings, especially with regards to hope, grief and rage. Climate prophets were not only invested in truth telling actions, but in ‘futuring’ more hopeful alternatives in the fashion of biblical prophets (Brueggemann 2018): ‘it's not just about how do you go around being really critical, but actually what does a better system look like?’ (3003, CCA). A member of Operation Noah stated that:

I increasingly think [that] a prophet has gone to where we belong and is looking back. So, for me, my job is to say, we are destined for beauty and for God and for harmony and humanity, and for nature […] we're destined for here, of goodness, wholeness (2008, Operation Noah).

**FIGURE 4:**
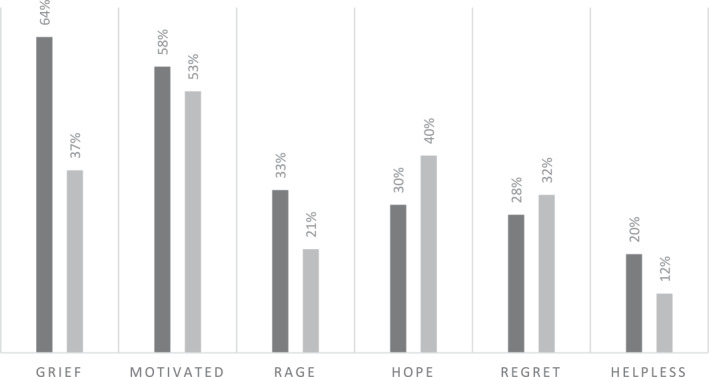
Three primary feelings reported in response to climate change. *Source:* ‘Religion, Theology and Climate Change’ Survey, 2023. Key: Black = Christian Climate Action and Operation Noah combined %; Grey = Manchester, Salford and Oxford Dioceses and CAFOD combined %.

Hope has been seen as a key part of the framing of secular climate activism, with figures like Greta Thunberg effective in its mobilisation (Svensson and Wahlström 2023; Molder et al. 2022; Kleres and Wettergren 2017). Similarly, Christian hope has also been established as an effective mobilising resource for climate activism (Bomberg and Hague 2018). Bock (2016) relates Christian hope to the prophetic role, arguing that a ‘critical hope’ is ultimately what propels Christians to climate activism: ‘critical’ of the systems that create the problem, and hopeful for an alternative future rooted in God's love. Interestingly, however, survey data revealed that in the groups where prophetic framings were most prevalent, respondents less frequently reported hope as one of their three primary feelings in response to climate change (Figure 4).

The shift in blame attribution to the system level and an awareness that system‐level change is required, produced less feeling of hope in CCA and Operation Noah. Put together, big problems with system origins can feel particularly impenetrable to collective action. In this context, some participants relocated hope in the building of a better system in the aftermath of climate catastrophe, where religious faith gives hope that ‘something else can emerge, even though things look crazy, something else can happen’ (1002, CCA). Losing hope in prevention of climate catastrophe, participants stated that ‘I have to think about hope in another way. And I now think this is more about […] shaping of the world to come (3002, CCA), because ‘what if climate change is […] the biggest kind of opportunity we have to change the way the system works?’ (3003, CCA).

Figure 4 also shows that members of CCA and Operation Noah more frequently expressed grief and rage as one of their three primary feelings in response to climate change. This finding supports Brueggemann's portrayal of the biblical prophet, who uses the rhetoric of grief to cut through the ‘numbness and denial’ of the unjust system. Brueggemann (2018, 46) states that ‘grief and mourning, that crying in pathos, is the ultimate form of criticism, for it announces the sure end of the [system]’. Grief was often articulated by participants as a response to becoming fully aware of the enormity of climate change (Malcolm 2025), with rage rooted in the recognition of an unjust system responsible for it.

## 6 Conclusion

The resurgence of the political wing of Christian climate activism has brought with it important nuances not reflected in existing analyses. By examining the rich variety of climate activisms across church dioceses, Christian aid organisations, and Christian social movements, we found that the political wing diverges from familiar stewardship framings, raising disputes over diagnostic framing (blame attribution) and prognostic framing (strategy and tactics). Reframed as ‘climate prophets’, Christians in social movement contexts interpreted their role as one of calling out an unjust system headed in the wrong direction, telling truth to power, and engaging in political protest tactics squarely aimed at ruffling system feathers. It is of global significance to the environmental movement that in certain enclaves and across denominations, Christian beliefs are being reinterpreted in ways that not only lead to the lifestyle activism of ‘climate stewards’, but to the political protest of ‘climate prophets’.

We must exercise some caution, however, in categorising different Christian groups in terms of attachment to the stewardship *or* prophetic framings. These categorisations may work well as ideal types, but in practice they are too neat. As critics have noted, frames do not exist as static cognitive maps that activists must choose between (Steinberg 1998). Instead, we need to talk about fram*ing* as an interactive process, involving multiple contestations in which the question of how to interpret situations and respond to them is open ended (Snow and Vliegenthart 2023). Stewardship was worked with across all groups, and even though it was the *dominant* interpretative framework at diocese level, it was being contested in exactly what it meant and how useful it was, as well as co‐existing with justice ideas (see also, Kidwell et al. 2018). Prophetic framings may have been the *dominant* interpretative framework in the Christian movement groups, but even they reluctantly affirmed stewardship as a default Christian language. Appeals to the prophetic role of Christians could also be found amongst church dioseces and CAFOD, albeit far less strongly. It should further be noted that participants were often not just members of one Christian group—social ties and group memberships were cross‐cutting, and so, therefore, were ideas.

Positions regarding activism of any type are also, of course, open to change. This is necessarily part of the ongoing nature of framing processes, and it is also because interpretations become reformulated through the experience of activism itself. As one participant put it, ‘it is not just a theological argument that will win them over’, it is also about experiences (4018, CAFOD). People move position as they participate in events, they exit activism, they escalate, they change their mind. As the member of CAFOD earlier reminded us: ‘I don't think I am at the moment [*a ruffler*], but things could change’. These possibilities for changing positions are the very stuff of political mobilisation.

Our findings also showed some challenges within Christian groups to the anthropocentric discourse that has been held partly responsible for the failings of institutional religion to fully engage with the climate change cause (Taylor 2010; White 1967). All groups contained some critique of stewardship for continuing to place humans above and in control of nature. Participants, at times, showed signs of developing a more biocentric outlook, where God is seen as within nature rather than separate from it (Malcolm and Scott 2024; Nita 2013, 236; Jantzen 1984). Yet, a different language to stewardship had not yet developed. One participant captured well what was a common response to the inadequacies of stewardship: ‘I think we need a different word. We need a different word there and I don't know what it is’ (1002, CCA). The absence of an alternative language to think about Christian climate activism in non‐anthropocentric terms perhaps explains something of the stickiness of stewardship as a term of reference for Christian groups. It is important therefore to take seriously the idea that interpretative framing is a task embedded within a wider cultural (and religious) discourse, which shapes and constrains the possibilities for meaning making (Snow 2008; Steinberg 1998).

Interestingly, the prophetic framing did not provide an alternative here either. It also could not break free from anthropocentric discourse in the sense that it pitched climate change concern in the language of social justice. Framings of environmentalism in terms of social justice have been critiqued for attachment to anthropocentric discourse because they translate ecological issues into issues that matter primarily for their human consequences, and thus remain human‐centred (Benford 2005). Importantly though, what is also signalled by our findings is that the anthropocentric discourse of Christian climate activists ‐ whether stewards or prophets ‐ does not appear to be a barrier to their motivation despite some existing literature (e.g., Taylor 2010) insisting that Christianity must become less anthropocentric if it is to become “greener”. Re‐enchanting the study of climate change activism not only therefore provides a fresh perspective on the truth telling nature of contemporary climate action, but advances our understanding of the multiple ways in which groups attached to insitutional religious faith mobilise against climate change.

## Funding

This research has been funded by the Arts and Humanities Research Council (AHRC), grant number: AH/W004089/1 ‘Religion, Theology and Climate Change’, Peter Scott, Celia Deane‐Drummond, Gemma Edwards and Finlay Malcolm.

## Ethics Statement

The ethics of this project were approved by the University of Manchester Research Ethics Committee, application reference 2023–15649‐27381.

## Conflicts of Interest

The authors declare no conflicts of interest.

## Data Availability

The data that support the findings of this study are available on Figshare: https://doi.org/10.48420/30234424 (interviews) and https://doi.org/10.48420/30234109 (survey).

## Endnotes

^1^
Christian enviornmentalism has been criticised for a lack of racial diversity in a similar way to the wider climate movement, see https://www.churchtimes.co.uk/articles/2022/1‐july/features/features/why‐is‐the‐green‐movement‐so‐white. There have been attempts to decolonise the climate change conversation among Christians, see Christian Aid: https://www.christianaid.org.uk/get‐involved/campaigns/climate‐change/climate‐and‐racial‐justice.

^2^

https://christianclimateaction.org/2023/06/09/sacrament‐of‐civil‐disobedience‐book‐club/.

^3^

https://www.operationnoah.org/bright‐now‐campaign/church‐divestment/.

^4^
The Charity Commission sets out regulations around the political activity of charities, see https://www.gov.uk/government/publications/speaking‐out‐guidance‐on‐campaigning‐and‐political‐activity‐by‐charities‐cc9/speaking‐out‐guidance‐on‐campaigning‐and‐political‐activity‐by‐charities. Maria Nita (2016, 214) suggests that,‘often religious activists are constrained on how political they can get by the charity status of the organisations they belong to’.

^5^
CAFOD, for example, campaign with farmers in developing countries to support seed sovereignty in the face of the agricultural policies of The World Bank: https://cafod.org.uk/campaign/fix‐the‐food‐system. Some appeals to the prophetic role are also made in other Christian aid groups, see https://www.christianaid.org.uk/get‐involved/campaigns/propheticactivist.
